# Filamin A phosphorylation by Akt promotes cell migration in response to arsenic

**DOI:** 10.18632/oncotarget.3617

**Published:** 2015-04-03

**Authors:** Lingzhi Li, Yongju Lu, Paul M. Stemmer, Fei Chen

**Affiliations:** ^1^ Department of Pharmaceutical Sciences, Eugene Applebaum College of Pharmacy and Health Sciences, Wayne State University, Detroit, MI 48201, USA; ^2^ The Proteomics Core and Institute of Environmental Health Sciences, School of Medicine, Wayne State University, Detroit, MI 48201, USA

**Keywords:** arsenic, Akt, filamin A, migration, patient survival

## Abstract

We had previously reported that trivalent arsenic (As^3+^), a well-known environmental carcinogen, induces phosphorylation of several putative Akt substrates. In the present report, we characterized one of these substrates by immunoprecipitation and proteomics analysis. The results indicate that a cytoskeleton remodeling protein, filamin A, with a molecular weight around 280 kDa, is phosphorylated by Akt in HEK-293 cells treated with As^3+^, which was also confirmed in human bronchial epithelial cell line, BEAS-2B cells. Additional biochemical and biological studies revealed that serine 2152 (S2152) of filamin A is phosphorylated by activated Akt in the cells treated with As^3+^. To further confirm the importance of Akt-dependent filamin A S2152 phosphorylation in As^3+^-induced cell migration, we over-expressed either wild type filamin A or the mutated filamin A in which the S2152 was substituted with alanine (S2152A). The capability of cell migration was reduced significantly in the cells expressing the mutated filamin A (S2152A). Clinically, we found that increased expression of filamin A predicts poorer overall survival of the lung cancer patients with adenocarcinoma. Thus, these data suggest that Akt dependent filamin A phosphorylation is one of the key events in mediating As^3+^-induced carcinogenesis. Antagonizing Akt signaling can ameliorate As^3+^-induced filamin A phosphorylation and cell migration, which may serve as a molecular targeting strategy for malignancies associated with environmental As^3+^ exposure.

## INTRODUCTION

Arsenic is a well-established carcinogen involved in the development of several types of cancers, e.g., cancers of the lung, skin, bladder, and liver [1]. Based on their chemical characteristics, the arsenic-containing compounds can be classified into organic and inorganic forms. It is believed that the inorganic form, especially, the trivalent arsenic (As^3+^), is much more toxic and carcinogenic. The International Agency for Research on Cancer (IARC) has classified arsenic as group I carcinogen [2]. As a naturally deposited metalloid, arsenic is widely distributed throughout the Earth's crust. Some common environmental conditions, such as aquifers under strongly reducing conditions in wet and flat regions and aerobic alkaline conditions in closed basins in arid and semiarid regions, can promote the release of arsenic from sediments to the dissolved forms in ground or drinking water [3]. Accumulating evidence had indicated an increased incident rate of lung cancer among populations with moderate to high level of As^3+^ exposure [4]. Worldwide, there is an estimation of 160 million people who are living in regions with elevated levels of arsenic in drinking water, including areas in the USA, China, Taiwan, Mexico, Mongolia, Argentina, India, Chile, and Bangladesh [5]. Thus, understanding how arsenic exposure is linked to human cancers is urgently needed.

Several lines of evidence suggest that arsenic, especially As^3+^, is capable of inducing malignant transformation of the normal cells by mechanisms of oxidative stress, DNA damage, interference of the DNA repairing processes, and disruption of the immune surveillance function [6]. In human bronchial epithelial cells or lung cancer cells, we had shown that As^3+^ is able to activate protein kinase Akt through either inducing miR-190 or initiating the signaling cascade from JNK to STAT3 that contributes to Akt-dependent EZH2 phosphorylation, cell transformation and/or migration [7–10]. Akt has long been viewed as a master regulator of epithelial-mesenchymal transition (EMT), cancer cell migration, invasion, and metastasis [11, 12]. Akt can directly phosphorylate ACAP1, Pak1, POSH, Girdin, and Twist that contain R-X-R-X-X-S/T or R-X-X-S/T Akt phosphorylation motif, followed by integrin recycling, actin cytoskeleton remodeling and EMT [13, 14].

Metastasis, in which cancer cells migrate to the secondary site(s) from their original tumor site, occurred in almost all cancers that progressed to later stages [15, 16]. In addition to the capabilities of cancer cells in proliferation and apoptotic responses for colonization at the secondary site, the key event of metastasis is migration of the cancer cells [17]. A number of cytoskeleton remodeling proteins are involved in cancer cell detachment, polarization and mobilization. In non-muscle cells, filamin A was the first discovered actin-filament crosslinking protein that interacts with integrins and several other cytoskeleton remodeling proteins [18–20]. Intriguingly, this protein has also been confirmed as a substrate for some protein kinases, including FAK, Lck [21], PAK1 [22], MAPK [23], and Akt [24], indicating that phosphorylation of filamin A is an essential step in initiating filamin A interaction with the cytoskeleton remodeling proteins or integrins for cell migration [25].

In this report, we provide evidence suggesting that As^3+^-induced cell migration is achieved through ROS-dependent activation of Akt that phosphorylates filamin A at serine 2152 in human bronchial epithelial cell line, BEAS-2B. Pharmacological inhibition of Akt or gene silencing of filamin A attenuated migration of the cells treated with As^3+^. These data, thus, may provide a new insight into the mechanism of tumorigenesis related to environmental As^3+^ exposure.

## RESULTS

### Identification of filamin A as a substrate of As^3+^-induced Akt

**Figure 1:**
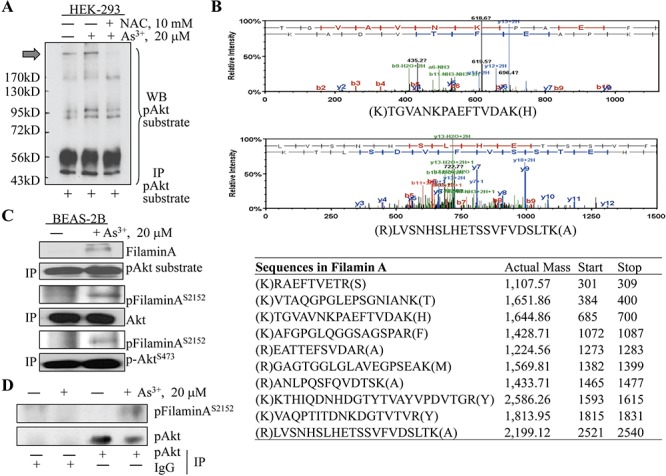
Identifying filamin A as an Akt substrate **A.** HEK-293 cells were treated with 20 μM As^3+^ in the presence or absence of 10 mM N-acetyl L Cysteine for 2 h. Cell lysates were prepared using non-denature buffer and subjected to immunoprecipitation (IP) using anti-Akt substrate antibody. Block arrow denotes protein band on the PVDF membrane that was retrieved for mass spectrometry analysis. **B.** Mass Spectrometry result shows the peptide profiling that overlaps sequence of filamin A. Top two panels are representative spectrums of the peptides from filamin A. **C.** BEAS-2B cell were treated as in (A), and subjected to IP using the indicated antibodies followed by Western blotting (WB). **D.** BEAS-2B cells were treated with 20 μM As^3+^ for 2 h, followed by IP using IgG or pAkt antibody as indicated and then WB using phospho-Filamin A (pFilamin A) or pAkt antibody.

We had previously demonstrated several Akt phosphorylation substrates in immunoprecipitation (IP) analyses of the BEAS-2B cells treated with As^3+^ [10]. To test whether these Akt substrates are also presented in other types of cells in response to As^3+^, we performed IP analysis using cell lysates from HEK-293 cells cultured in the absence or presence of 20 μM As^3+^ for 2 h and an antibody specifically recognizing the phosphorylated Akt phosphorylation motif, R-X-R-X-X-S/T. As depicted in Fig. 1A, a band with an estimated molecular weight about 280 kDa was constitutively detected in HEK-293 cells (pointed with a black block arrow). Treatment of the cells with As^3+^ enhanced the density of this band considerably. Pre-incubation of the cells with an antioxidant, NAC that scavenges reactive oxygen species (ROS), diminished this band completely, suggesting that the occurrence of this band is ROS-dependent. To determine the nature of this protein band that can be recognized by an anti-Akt phosphorylation substrate antibody, we retrieved this band from Western blotting membrane and subjected it to proteomics analysis through tryptic digestion and peptide identification using orbitrap Fusion mass spectrometry as described under Materials and Methods. The peptides identified from this analysis suggested a possible presence of 11 proteins in this protein band as detected in IP, among which only two proteins, filamin A and inositol 1,4,5-trisphosphate receptor type 3 (ITPR3), are within the range of molecular weight between 200 and 300 kDa where the original band was positioned. The filamin A was represented by 10 peptides, whereas ITPR3 was represented by only 4 peptides. Thus, we concluded that the most abundant protein in this IP band is filamin A based on the assumption that the number of peptides identified in mass spectrometry is generally proportional to its abundance or concentration in the sample.

### Protein-protein interaction between Akt and filamin A

To further confirm filamin A as an Akt substrate, we next determined whether there is a direct interaction between protein kinase Akt and filamin A in BEAS-2B cells treated with As^3+^ through immunoprecipitation. Again, the anti-Akt substrate antibody was able to pull down filamin A in the cells treated with As^3+^ (Fig. 1C, top two panels). Using antibodies against either the total Akt or phospho-Akt (pAkt), we showed that both activated and total Akt were able to interact with filamin A that was phosphorylated at serine 2152 (S2152), and this interaction occurred only in the cells treated with As^3+^ but not the cells without As^3+^ treatment (Fig. 1C). To exclude the possibility of non-specific protein-protein interaction during IP, we also used control IgG in our immunoprecipitation assay. It is clear that only the anti-pAkt antibody, but not IgG, can pull down the phosphorylated filamin A (Fig. 1D). Therefore, these data provided substantial evidence indicating that filamin A is an endogenous Akt substrate that can be phosphorylated by Akt through direct interaction.

### ROS mediate As^3+^-induced Akt activation that phosphorylates filamin A

**Figure 2:**
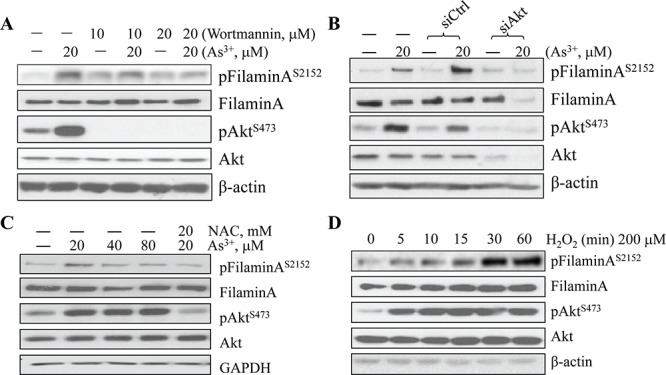
As^3+^-induced Akt activation and filamin A phosphorylation are ROS dependent **A.** BEAS-2B cells were treated with 20 μM As^3+^ for 2 h with or without pretreatment with 20 μM wortmannin. The protein levels of phospho-filamin A^S2152^, filamin A, pAkt^S473^, Akt, and β-actin were determined by WB. **B.** BEAS-2B cells were transfected with 50 nM Ctrl siRNA or Akt siRNA with or without an additional treatment of As^3+^ for 2 h. WB was then performed as in (A). **C.** BEAS-2B cells were treated with the indicated concentrations of As^3+^ and/or NAC. WB was then performed as in (A). **D.** Time-dependent induction of filamin A phosphorylation by H_2_O_2_ in BEAS-2B cells.

To extend above observations, we pretreated the cells with Wortmannin, an inhibitor that is relatively specific for the upstream kinase, PI3K that phosphorylates and activates Akt, in the BEAS-2B cells. A roughly dose-dependent inhibition of the As^3+^-induced filamin A phosphorylation was observed in the cells pretreated with 10 or 20 μM Wortmannin that diminished Akt activation completely (Fig. 2A). This inhibitor had no detectable effect on the protein levels of non-phospho-filamin A, total Akt or β-actin (Fig. 2A). The requirement of activated Akt kinase in As^3+^-induced filamin A phosphorylation was further validated by silencing Akt using siRNA that specifically down-regulates Akt expression. As indicated in Fig. 2B, the control siRNA with scramble sequence (siCtrl) has no inhibitory effect, neither on the level of phospho-filamin A (pFilamin A^S2152^) nor the total Akt. Silencing Akt using Akt specific siRNA (siAkt) not only reduced the levels of total Akt and the activated pAkt, but also attenuated phosphorylation of filamin A in the cells treated with As^3+^ (Fig. 2B). Taken together, these data clearly suggest that the As^3+^-induced Akt activation, as determined by the phosphorylation on serine 473 (S473), is essential for the phosphorylation of filamin A at S2152.

We had previously demonstrated that As^3+^ induces excessive generation of ROS that contribute to Akt activation [10] and the subsequent transformation of the cells [26]. To address the role of ROS in As^3+^-induced filamin A phosphorylation, we first treated the cells with As^3+^ in the absence or presence of 20 mM NAC, a widely used ROS scavenger. As expected, NAC prevented As^3+^-induced Akt activation and filamin A phosphorylation (Fig. 2C). The role of ROS in As^3+^-induced filamin A phosphorylation was additionally confirmed by treatment of the BEAS-2B cells with 200 mM H_2_O_2_ for 5 to 60 min. Indeed, H_2_O_2_ is able to induce Akt activation and filamin A phosphorylation in a time dependent manner. The activation of Akt and phosphorylation of filamin A can be detected as early as 5 min after the cells treated with H_2_O_2_. At 30 min, the filamin A phosphorylation reached plateau (Fig. 2D).

### As^3+^ induces cell migration through Akt activation

**Figure 3:**
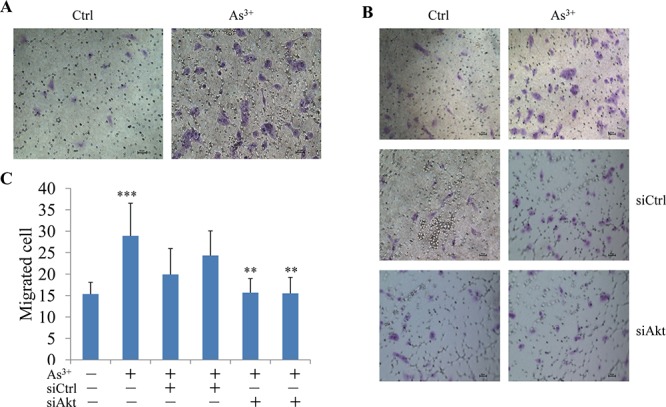
As^3+^-induced cell migration is Akt dependent **A.** Migration assay shows elevated motility of the BEAS-2B cells treated with 20 μM As^3+^ for 24 h. **B.** Silencing Akt by siRNA reduces As^3+^-induced cell migration. BEAS-2B cells were transfected with 50 nM control siRNA (siCtrl) or Akt siRNA (siAkt). **C.** Semi-quantification of the migrated cells as shown in (B). Data are expressed as mean ± SD, *n* = 12, ***p < 0.001, **p < 0.01.

Activation of Akt has been linked to a number of cellular processes, including cell growth, anti-apoptosis, transformation, migration, invasion, and metastasis of the cancer cells [27]. Accordingly, it is plausible to hypothesize that the activation of Akt by As^3+^ can be involved in cell migration. To test this, we first treated BEAS-2B cells with As^3+^ and then measured cell migration capability using Matrigel-based Migration Chambers. As^3+^ treatment promoted cell migration significantly (Figs. 3A and 3C). Silencing Akt using Akt specific siRNA reduced the number of the migrated cells in response to As^3+^. As^3+^-induced cell migration was not affected by the control siRNA (siCtrl, Figs. 3B and 3C).

### Filamin A phosphorylation is required for As^3+^-induced cell migration

**Figure 4:**
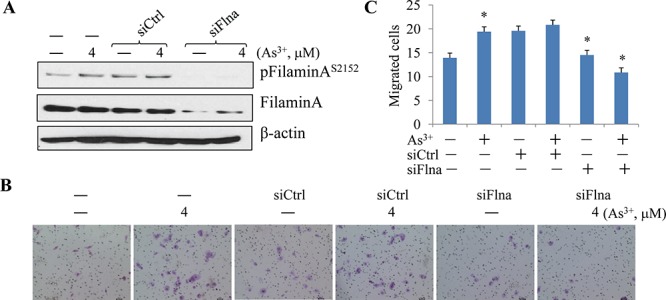
Silencing filamin A prevented As^3+^-induced cell migration **A.** BEAS-2B cells were transfected with 50 nM control siRNA (siCtrl) or filamin A siRNA (siFlna) with or without treatment by 4 μM As^3+^ for 24 h. **B.** Representative images of the migration assays. BEAS-2B cells were treated as in (A). **C.** Semi-quantification of the migrated cells as shown in (B). Data are expressed as mean ± SD, *n* = 12, *p < 0.05.

To answer whether filamin A phosphorylation served as a downstream signal for As^3+^-induced Akt activation that facilitates cell migration, we next silenced filamin A by transfection of the cells with filamin A specific siRNA (siFlna) followed by measuring the cell migration. Filamin A specific siRNA, siFlna, reduced the levels of both filamin A and the S2152-phosphorylated filamin A (pFilamin A^S2152^) notably (Fig. 4A). The control siRNA, siCtrl, exhibited no significant effects on these proteins, neither pFilamin A, nor total Filamin A. In cell migration assays, again, As^3+^ increased the number of migrated cells significantly (Figs. 4B and 4C). No inhibition of the As^3+^-induced cell migration was detected in the cells transfected with the control siRNA (siCtrl). In contrast, a strong inhibition of cell migration induced by As^3+^ was observed in the cells transfected with siFlna that specifically silences filamin A (Figs. 4B and 4C). These observations, thus, not only reinforced the notion that filamin A might be an important regulator for cell migration [24, 28], but also provided strong evidence indicating that filamin A is a downstream effecter to bridge As^3+^-induced Akt activation and migration.

### Disruption of filamin A phosphorylation impairs cell migration

**Figure 5:**
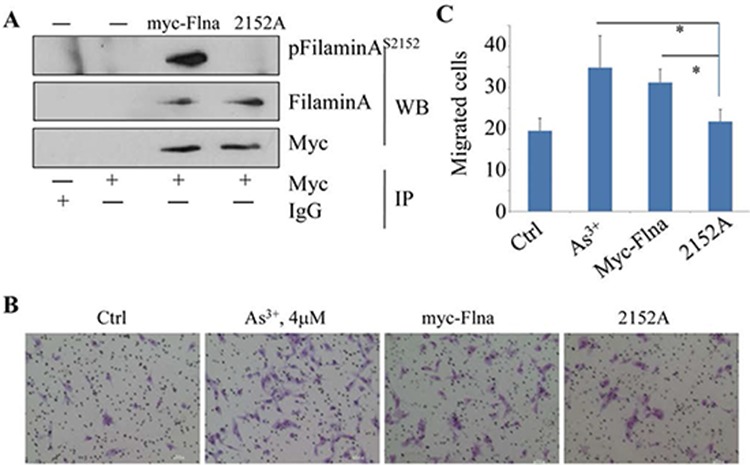
Phosphorylation of Filamin A on Ser2152 is required for As^3+^-induced cell migration **A.** BEAS-2B cells were transfected with 2 μg myc-filamin A (wild type, myc-Flna) plasmid or myc-filamin A S2152A (2152A) plasmid, followed by IP using anti-myc tag antibody and WB using the indicated antibodies. **B.** Representative images of cell migration assay of the BEAS-2B cells that were transfected with 2 μg myc-filamin A (myc-Flna) plasmid or myc-filamin A S2152A (2152A) plasmid. **C.** Semi-quantification of the migrated cells as shown in (A & B). Data are expressed as mean ± SD, *n* = 12, *p < 0.05.

Several lines of evidence had indicated the essential role of filamin A for mammalian cell locomotion. However, how this function of filamin A is regulated remains to be fully understood. Since As^3+^ induces direct interaction between Akt and filamin A (Fig. 1), which leads to S2152 phosphorylation of filamin A (Figs. 2A and 2B), it is very likely that the migration regulation by filamin A depends on the phosphorylation of filamin A at S2152. To explore this possibility, we transfected BEAS-2B cells with an expression vector containing myc-tagged full-length filamin A (myc-Flna, wild-type) or myc-tagged filamin A in which the serine 2152 was mutated to alanine (S2152A). The substitution of S2152 with alanine abrogated the phosphorylation of filamin A as revealed in an IP experiment using anti-myc tag antibody (Fig. 5A). Resembling the cells treated with As^3+^, cells with an overexpression of the full-length wild-type filamin A exhibited an enhanced migration capability (Figs. 5B and 5C). On the other hand, the migratory ability of the cells expressing the mutated filamin A, S2152A, was much reduced relative to the cells expressing wild-type filamin A (Figs. 5B and 5C). Thus, phosphorylation of filamin A at S2152 by As^3+^-activated Akt is a prerequisite step for cell migration.

### The expression level of filamin A predicts prognostic outcomes of the lung cancer patients

**Figure 6:**
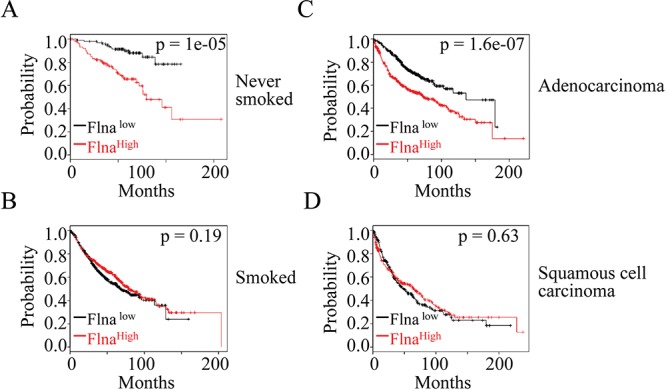
The expression level of filamin A is a prognostic factor for the lung cancer patients **A & B.** High level of filamin A predicts poorer overall survival of the lung cancer patients who were never-smoked, but not those who were smoked. **C & D.** High level of filamin A predicts poorer overall survival of the patients with adenocarcinoma, but not those patients with squamous cell carcinoma. Statistical significance was determined by logrank *p*-values as indicated. The panels depict Kaplan-Meier survival probability with the probe ID 200859_x_at. Similar data were obtained when probe ID 214752_x_at and 213746_s_at were used.

To establish clinical relevance of the above findings, we investigated correlation between the expression level of filamin A and the survival times of the lung cancer patients by using an online database of Kaplan-Meier Plotter that contains gene profiling information from 2,437 human lung cancer samples [29]. Three overlapping sets of probes are included in this database to mostly detect the open-reading-frame (ORF) of the filamin A mRNA. In initial analysis, we found that the expression level of filamin A did not distinguish poorer or better survival times of the patients when all types of lung cancers were included. After stratifying the lung cancer patients based on their status of cigarette smoking, e.g., never-smoked or smoked, it was found that higher expression level of filamin A predicts poorer overall survival of the patients who were never-smoked (Fig. 6A). In contrast, higher expression of filamin A appeared to be no effect on the overall survival among those patients who were current or former smokers (Fig. 6B). Since it had been well-documented that cigarette smoking contributes to different histological subtypes of the lung cancer [30], we believed that the opposite predictive power of filamin A expression between smoked and never-smoked might be a reflection of the different histological subtypes of the lung cancers. Therefore, we next compared the overall survival of the patients with either adenocarcinoma or squamous cell carcinoma based on the higher or lower expression level of filamin A. It is true that higher level of filamin A predicts a significant poorer survival of the patients with adenocarcinoma (Fig. 6C), but not the squamous cell carcinoma (Fig. 6D).

## DISCUSSION

The molecular or biochemical basis of As^3+^-induced cell transformation, carcinogenesis and tumorigenesis is not well defined. In the present study, we unraveled a signaling cascade for As^3+^-induced cell migration. In both human HEK-293 cells and bronchial epithelial cell line BEAS-2B cells, our data suggest that As^3+^ induces activation of Akt that interacts with and phosphorylates filamin A, an actin-binding protein responsible for crosslinking actin filaments into the membrane glycoproteins and integrins [18–20]. Activation of Akt by As^3+^ appears to be ROS dependent, based on the fact that NAC, a widely used antioxidant, blocked As^3+^-induced Akt activation and the subsequent filamin A phosphorylation. This notion was additionally supported by the observation that H_2_O_2_, the most common intracellular oxidative molecule, can activate Akt, leading to the phosphorylation of filamin A. Silencing either Akt or filamin A using specific siRNAs impaired cell migration in response to As^3+^. Furthermore, our data also demonstrated that Akt-dependent phosphorylation on S2152 of filamin A is involved in cell migration, since overexpression of the mutated filamin A, in which S2152 was replaced with alanine, reduced the capability of cell migration. Taken together, these results provide the first evidence indicating that As^3+^ induces cell migration through signaling pathway from ROS to Akt that phosphorylates filamin A.

The association between environmental As^3+^ exposure and carcinogenesis had been well-established. Majority of studies so far addressed the potential effects of As^3+^ on the initiation of cell transformation and/or carcinogenesis, such as DNA damage responses, inhibition of the DNA repairing machinery, impairment of the immune surveillance system, alteration of the epigenetic modifications on the genome, and activation of a wide range of signaling pathways that linked to transcription factors and gene expression [4]. Very limited information is available, however, on whether As^3+^ is also capable of regulating the key processes of tumorigenesis, including tumor cell motility, invasion and metastasis. Several reports as well as our previous studies had suggested that As^3+^ is a potent activator for the multi-functional protein kinase Akt that had been linked to tumor angiogenesis, tumor cell metastasis and metabolism [7–10, 31, 32]. Accumulating evidence has suggested that Akt can not only provide the cells with growth advantages by eliciting proliferative or anti-apoptotic signals but also engage with motility of the cells through regulating actin tethering and stress fiber assembly [33]. Thus, it is very likely that in addition to the earlier events of carcinogenesis, As^3+^ can also participate in the tumorigenic process through Akt activation.

As revealed by IP using anti-Akt phospho-substrate antibody followed by proteomic analysis of the proteins in the immunocomplexes, we provide the first evidence demonstrating a direct interaction between Akt and filamin A in cellular response to As^3+^. This interaction results in phosphorylation of filamin A at S2152. Akt-dependent phosphorylation of filamin A at S2152 has been previously determined in prostate cancer cells and the IGF-1-treated MCF-7 cells [24, 34]. It is believed that this phosphorylation can prevent cleavage of filamin A and enhance its association with caveolin-1 [24, 34]. Few studies also implied that S2152 phosphorylation of filamin A by other protein kinases, such as PAK1 or RSK, might be involved in the formation of membrane ruffling and lamellipodia extension, the necessary steps for cell migration and tumor cell metastasis [35, 36]. Furthermore, studies using molecular dynamics simulations unraveled that phosphorylation of filamin A at S2152 can facilitate integrin binding of filamin A by conformational changes that open the primary binding site of integrin on the Ig repeat 21 of filamin A [25]. Thus, an Akt-dependent phosphorylation of filamin A induced by As^3+^ may enhance the capability of integrin binding of filamin A, and consequently, foster migration of the cells.

The observation that increased filamin A expression predicts poorer overall survival of the never-smoked patients and adenocarcinoma (Fig. 6) provided compensatory supporting evidence suggesting involvement of filamin A in lung malignancy. More importantly, it is highly possible that this observation serves as a strong indication that this protein may impact the aggressiveness of the specific histological subtypes of the human lung cancer. Many earlier studies had revealed that adenocarcinoma most likely occurred in the younger, never-smoked and females with a much aggressive disease progression [37, 38]. It is also noteworthy that there is an increased proportion of adenocarcinoma among U.S. copper smelter workers who were exposed to As^3+^ through inhalation [39]. Due to the higher frequency of mutation on the gene loci encoding LKB1, an extensively studied tumor suppressor that suppresses metastasis of the tumor cells, adenocarcinoma appears to be more metastatic compared with squamous cell carcinoma or large cell carcinoma [40, 41]. As tumor cell metastasis is one of the major biological factors contributing to the aggressiveness of tumors, it is plausible, thus, to assume that increased level of filamin A can enhance the migration and metastasis potentials of the cancer cells, especially for the adenocarcinoma. Although phosphorylation status of filamin A on patient prognostic outcomes was not evaluated due to the unavailability of such data, a proportional increase of the phosphorylated filamin A can be envisioned among those patients with a higher level of overall expression of filamin A. Accordingly, the prediction of poorer survival of the never-smoked lung cancer patients with adenocarcinoma is in great agreement with our experimental findings showing As^3+^ induces Akt-dependent phosphorylation of filamin A and cell migration.

In summary, this report presented a novel mechanism for the contributions of As^3+^ to human cancer. The data suggest that As^3+^ is capable of regulating the tumorigenesis through ROS dependent activation of protein kinase Akt that phosphorylates filamin A, leading to an enhanced crosslinking between actin filaments and integrin by filamin A and the subsequent migration of the cells. As cell motility or migration is one of the key steps for tumor cells metastasis [42], regulation on the binding capability of filamin A on integrin and actin network certainly will influence the disease progression of the cancer patients. Indeed, a recent study showed that filamin A promotes migration and invasive potential of breast cancer cells through interaction with cyclin D1 [43]. This notion was further supported by the observations that overexpression of filamin A is associated with highly metastatic cancers, such as melanoma [44], neuroblastoma [45], breast cancer [46], and prostate cancer [47]. Accordingly, the findings in this report may be potentially translated into new therapeutic strategies by targeting Akt kinase and filamin A in cancers resulting from exposure to As^3+^ or other environmental carcinogens.

## MATERIALS AND METHODS

### Cell lines and reagents

The human bronchial epithelial cell line BEAS-2B, and human embryonic kidney 293 cell line HEK-293 were obtained from the American Type Culture Collection (ATCC, Manassas, VA). The BEAS-2B cells were cultured in Dulbecco's modified Eagle's medium (DMEM, Invitrogen, Grand Island, NY) containing 5% fetal bovine serum (FBS, Invitrogen), 1% penicillin/streptomycin, and 1% L-glutamine (Sigma, St. Louis, MO) at 37°C humidified incubator with 5% CO_2_. The human embryonic kidney 293 cell line HEK-293 was maintained in DMEM containing 10% FBS, 1% penicillin/streptomycin, and 1% L-glutamine at 37°C humidified incubator with 5% CO_2_. The cells were treated with As^3+^ [arsenic (III) chloride, Sigma-Aldrich, St. Louis, MO], with the indicated concentrations and times when the cells reached an approximately 80% confluence. In some experiments, PI3K/Akt inhibitor Wortmannin or ROS scavenger N-acetyl-cysteine (NAC) were added 2 h prior to As^3+^ treatment.

### Plasmid preparation and transfection

Constructs of pcDNA3-myc-Flna wild type (WT) and pcDNA3-myc-Flna S2152A were purchased from Addgene (Cambridge, MA). The plasmid DNA was amplified in DH5α Competent cells (Invitrogen, Grand Island, NY) and purified using either Plasmid Purification Kit (QIAGEN, Valencia, CA) or HQ Mini Plasmid Purification Kit (Invitrogen) according to the manufacturer's protocol. Cells in 6-well plates were transfected either with 50 ng plasmid DNA using Lipofectamine RNAiMAX Transfection Reagent (Invitrogen) or 2 μg plasmid DNA with Nucleofector (Lonza, Anaheim, CA) with program G016.

### Western blotting

For regular Western blotting, the cell lysates were prepared by RIPA cell lysis buffer (Cell Signaling, Danvers, MA) followed by ultrasonication and centrifugation. The supernatants were aspirated and proteins were quantified using a SpectraMax spectrophotometer (MDA Analytical Technologies). LDS sample buffer (Invitrogen) and dithiothreitol were added before denature. The samples were separated on 7.5% SDS-PAGE running gel and then transferred to PVDF membranes. The membranes were blocked in 5% non-fat milk in TBST and incubated with the indicated primary antibodies at 4°C overnight, and then incubated with second antibodies at room temperature for 1 h and washed with TBST 3 times for 10 min each. The protein levels were detected using CDP-Star Reagent (New England Biolabs, Ipswich, MA), SuperSignal West Pico (Thermo Fisher Scientific, Waltham, MA) or Immobilon Western chemiluminescent HRP substrate (MILLIPORE, Billerica, MA). The primary antibodies used in Western blotting include anti-phospho-Akt (Ser473) (Cell Signaling), anti-phospho-Akt substrate RXRXXS*/T* (Cell Signaling), anti-phospho-Filamin A (Cell Signaling), anti-Filmain A (Cell Signaling), anti-Akt (Cell Signaling), anti-myc tag, (Cell Signaling), anti-GAPDH (Cell Signaling), and anti-β-actin (Sigma).

### Immunoprecipitation (IP) and proteomics

After the indicated treatments, the cells were collected in IP lysis buffer and fragmented through passing the cells in a syringe equipped with 21.5 needle for 10–15 times. About 500 μg protein for each indicated sample was prepared and then incubated with the specific antibodies that were conjugated with agarose beads at 4°C agitation overnight. The immunocomplexes were pulled down by centrifugation at 3,000 rpm for 10 min and separated in SDS-PAGE gel for regular Western blotting. After extensive washes, the protein bands of interest on the PVDF membrane were retrieved with a scalpel followed by washing the membrane slices in double diluted water. Proteins on the membrane slices were digested overnight in 50 mM ammonium bicarbonate, 30% acetonitrile and sequencing-grade trypsin (Promega, Madison, WI) at 37°C. The digestion solution was collected and the membranes were washed 2 times with 70% acetonitrile (ACN) and 1% FA with sonication. The solutions were pooled, speed vacuumed to dryness, and solubilized in 2% ACN, 0.1% FA. Protein peptides were separated by reverse phase chromatography (Acclaim PepMap100C18 column, Thermo Scientific), followed by ionization with the Nanospray Flex Ion Source (Thermo Scientific), and introduced into an orbitrap Fusion mass spectrometer (Thermo Scientific). The obtained spectra were analyzed using Proteome Discoverer 1.4.0.288 (Thermo Scientific) which incorporates the Mascot algorithm (Matrix Science, Boston, MA).

### siRNA transfection

Cells with a concentration of 1 × 10^5^/ml were seeded into 6-well plates and reverse transfected with siRNAs using Lipofectamine RNAiMAX reagent (Invitrogen). For short term treatment, 20 μM As^3+^ was added at 24 h and cultured for an additional 2 h. SiRNA that specifically silence filamin A was purchased from Santa Cruz Technology (Santa Cruz, CA); control siRNA and siRNA specifically silencing Akt were purchased from Cell Signaling (Beverly, MA).

### Cell migration assay

Cell migration was determined using BD BioCoat^TM^ Matrigel^TM^ Migration Chambers (8 μm filter). BEAS-2B cells were seeded with a density of 1.5 × 10^4^/well (siRNA transfection) or 1 × 10^5^ /well (plasmid transfection) in the upper chamber. DMEM medium containing 5% FBS was added into the lower chambers. The chambers were incubated at 37°C for 4 to 6 h, followed by replacing the medium contain 0.5% FBS and 4 μM As^3+^. After 24 hrs incubation, the cells in the upper chambers were scrubbed out using cotton-tipped swab. The cells on the lower surface of the membrane were stained with the Diff-Quik Kit (Dade Behring). The migrated cells were counted under a microscope for 12 random fields in each group.

### Statistics

Microsoft Excel was used for statistical analyses of the quantitative data. The data are expressed as mean ± standard deviation (SD). The student's *t*-tests were used to determine the statistical significance of differences between samples treated under different conditions. Differences were considered statistically significant when *p < 0.05, **p < 0.01, or ***p < 0.001.
